# Mutation of putative N-Linked Glycosylation Sites in Japanese encephalitis Virus Premembrane and Envelope proteins enhances humoral immunity in BALB/C mice after DNA vaccination

**DOI:** 10.1186/1743-422X-8-138

**Published:** 2011-03-25

**Authors:** Yu Zhang, Puyan Chen, Ruibing Cao, Jinyan Gu

**Affiliations:** 1 Key Laboratory of Animal Diseases Diagnostic and Immunology, Ministry of Agriculture, College of Veterinary Medicine, Nanjing Agricultural University, Nanjing 210095, China

## Abstract

Swine are an important host of Japanese encephalitis virus (JEV). The two membrane glycoproteins of JEV, prM and E, each contain a potential N-linked glycosylation site, at positions N15 and N154, respectively. We constructed plasmids that contain the genes encoding wild-type prME (contain the signal of the prM, the prM, and the E coding regions) and three mutant prME proteins, in which the putative N-linked glycosylation sites are mutated individually or in combination, by site-directed mutagenesis. The recombinant plasmids were used as DNA vaccines in mice. Our results indicate that immunizing mice with DNA vaccines that contain the N154A mutation results in elevated levels of interleukin-4 secretion, induces the IgG1 antibody isotype, generates greater titers of anti-JEV antibodies, and shows complete protection against JEV challenge. We conclude that mutation of the putative N-glycosylation site N154 in the E protein of JEV significantly enhances the induced humoral immune response and suggest that this mutant should be further investigated as a potential DNA vaccine against JEV.

## 1. Introduction

Japanese encephalitis virus (JEV) belongs to the genus Flavivirus, and the genus Flavivirus include many clinically important pathogens, such as dengue virus (DENV), West Nile virus (WNV), yellow fever virus, Murray Valley encephalitis virus, St.Louis encephalitis virus, and tick-borne encephalitis virus (TBEV). Japanese encephalitis virus (JEV) mostly causes infection of the central nervous system in humans and equines and stillbirths in swine [1,2]. The virus is zoonotic, cycling between birds and mosquitoes, and is transmitted to humans by infected mosquitoes. Since swine serve as a reservoir and amplifier of the virus [3], the development of a swine vaccine against JEV is a high priority, as it could help prevent epidemics in humans.

JEV contains a single-stranded, plus-sense RNA genome of ~11 kb. It consists of a single open reading frame that codes for a large polyprotein of 3432 amino acids that is co- and post-translationally cleaved into three structural proteins (capsid, C; premembrane, prM; and envelope, E) and seven nonstructural proteins [4,5]. Envelope is the major structural protein, and makes up the surface of the avivirus particle. E protein has numerous neutralization epitopes, which mediate attachment to host cells, and a putative receptor-binding domain that induces the host immune response [6,7]. Though prM is able to fold independently of the E protein, correct folding of the E protein requires co-synthesis with prM [8]. PrM interacts with E to form prM-E heterodimers, which are important for the formation of immature virions[9,10], and the signal of the prM determine translocation and orientation of inserted protein, hence the topology of prM and E [11]. Therefore, the signal of the prM and the prM protein play an important role in maintaining its native conformation of E protein.

N-linked glycans of viral proteins play important roles in modulating the immune response. Glycans can be important for maintaining the appropriate antigenic conformations, shielding potential neutralization epitopes, and may potentially alter the proteolytic susceptibility of proteins [12,13]. In the JE viruses, the prM protein contains one putative N-linked glycosylation site, at N15. E protein also has one putative N-linked glycosylation site, at N154. Studies with JEV, TBEV and WNV have found that deletion of the N-linked glycosylation site in prM or E led to a decrease in virus release [14-16]. However, the effects of these putative N-linked glycosylation sites on the immune response to JEV remained elusive.

Our primary aim in this work was to investigate the role of the putative prME N-linked glycosylation sites in inducing an immune response. It is known that immunizing mice with plasmids encoding the prM and E glycoproteins of JEV provide varying degrees of protection against the virus [17]. In this study, we constructed plasmids containing both the wild-type prME and mutant prME genes, in which the N-linked glycosylation sites are mutated individually or in combination. The immunogenicity of the three prME glycosylation mutants was evaluated in mice. We determined that mutating N154 of prME significantly enhanced the immune response in mice and propose that this mutant should be explored as a swine vaccine against JEV.

## 2. Materials and methods

### 2.1 Cells and virus

The NJ2008 strain (GQ918133) of JEV was isolated from brain tissues of aborted fetuses of sows, which were obtained from a piggery in the Jiangsu province in 2008. The NJ2008 strain of JEV was propagated in baby hamster kidney (BHK-21) cells (ATCC CCL-10) for the plaque reduction neutralization test (PRNT) and challenge test. The supernatants of the infected cells were clarified and stored at -80°C for animal challenge. The viral titers of the supernatants were approximately 7.3 × 10^8 ^PFU as determined by the PRNT. Monkey kidney cells (Vero) (ATCC) used for recombinant plasmid transfection were grown and maintained in Dubach's modified Eagle's medium (DMEM) supplemented with 10% heat-inactivated fetal bovine serum, 100 μg/ml of streptomycin, and 100 μg/ml of penicillin.

### 2.2 Construction of site-directed prME mutants

**Figure 1:**
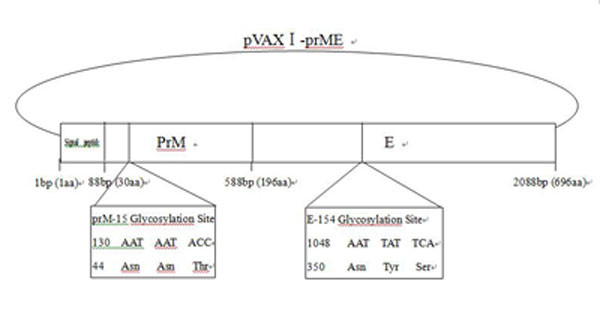
**Schematic representation of the recombinant JEV plasmid pVAXI -prME**. The **Y **symbol denotes the positions of the predicted glycans in the prM and E proteins. The amino acid sequences of the prM and E glycosylation sites are expanded at the bottom of the figure.

A single cDNA fragment containing genomic nucleotides (nt) 390 to 2478 was amplified by reverse transcriptase-mediated PCR (RT-PCR). The JEV forward and reverse primers were 5'-TACGAATTCATGGGCAGAAAGCAAAAC-3' and 5'-CATCTCGAGAGCATGCACATTGGTCGCTAA-3', respectively. Restriction enzyme sites for EcoRI and XhoI were engineered into the 5' and 3' ends of the cDNA, respectively. An in-frame translation termination codon was introduced upstream of the XhoI restriction site at the 3' terminus. Amplified cDNA was digested with EcoRI and XhoI and inserted into the EcoRI-XhoI site of the eukaryotic expression plasmid vector pVAXI (Invitrogen) (Figure 1). The Quikchange kit (Stragene) was used to mutate the putative glycosylation sites in the pVAXI-prME plasmid. The single mutants were referred to as pVAXI-prME-M1 (mutation at N15 site of prM) and pVAXI-prME-M2 (mutation at N154 site of E). The double mutant was referred to as pVAXI-prME-M3 (Table 1).

**Table 1:** pVAX-prME mutant vectors and predicted glycosylation patterns.

**Name**	**Mutation site**		**Glycosylation**	
	**prM-15**	**E-154**	**prM**	**E**
pVAX-prME-wt	Asn Asn Thr	Asn Tyr Ser	+	+
pVAX-prME-M1	**Ala Ɵ **Asn Thr	Asn Tyr Ser	-	+
pVAX-prME-M2	Asn Asn Thr	**Ala Ɵ **Tyr Ser	+	-
pVAX-prME-M3	**Ala Ɵ **Asn Thr	**Ala Ɵ **Tyr Ser	-	-

Amino acid residues of JEV prME protein targeted for mutagenesis. prME -M1 and prME -M2 are single site mutants, whereas prME -M3 is the double mutant. In all mutants, the Asn codon was replaced by an Ala codon in its N-glycosylation sites (N-X-T/S). Asterisks represent mutated N-glycosylation sites.

### 2.3 Transfection and Western blotting

BHK-21 cells were seeded at a concentration of 2.5 × 10^4 ^cells/well into 6-well tissue culture plate until the cells reached approximately 70-80% confluence. Transfection was performed with LipofectAMINE 2000 reagent (Invitrogen) as specified by the manufacturer. The transfected cells were collected at 48 h post-transfection and lysed in buffer L (50 mM Tris, 150 mM NaCl, 2 mM EDTA, 1% Triton X-100, 0.5 mM phenylmethylsulfonyl sulfate, pH 7.5). The lysates were centrifuged at 10,000 × g for 10 min to clear cellular debris and inactivated at 56°C for 1 h, and aliquots of each proteins were digested with 500 U of peptide N-glycosidase F (PNGase F, New England Biolabs) for 1 h at 37°C or mock digested as a negative control. Samples were then analyzed under denaturing conditions by 12% SDS-polyacrylamide gel electrophoresis (SDS-PAGE), and Western blotting was performed using JEV-positive serum (kept in our laboratory). Detection was performed using chemilumines.

### 2.4 Mouse immunization

Four-week old female BALB/c mice were purchased from the Animal Center of Nanjing Army Hospital, Nanjing, China. All mice were maintained in sterile cages in specific-pathogen-free environments. Five groups of mice (fourteen mice per group) were inoculated with one of the following plasmids: pVAX-prME-WT, pVAX-prME-M1, pVAXI-prME-M2, pVAX-prME-M3, and pVAX (control group). The mice received 50 μg of recombinant plasmids intramuscularly (IM) into each thigh (total dose 100 μg). All groups were inoculated three times at 2-week intervals. Serum samples were collected from the central tail vein before immunization on days 7, 21 and 35 after the prime immunization, and sera were stored at -20°C.

One week after the second boost immunization, four mice from each group were sacrificed and their spleens removed aseptically for in vitro splenocyte culture. The remaining mice were challenged by i.p. injection of a lethal dose of the JEV NJ2008 strain (5 × 10^6 ^PFU). Survival of the mice was monitored daily up to 15 days post-challenge. All animal experiments were conducted according to the guidelines approved by the Animal Ethical and Experimental Committee of the Nanjing Agricultural University.

### 2.5 ELISA assays to profile antibodies and measure cytokine production

Antibody levels were measured in sera were collected on days 7, 21 and 35 after the prime immunization. Antibody subtypes were analyzed by ELISA [18] with some modifications. The 96-well Maxi-sorpTM plates (Nunc) were coated overnight with purified JEV particles (10 ng/ml) in 0.1 M sodium carbonate (pH~9.5). The presence of IgG, IgG1 and IgG2a was measured using HRP-conjugated antibodies that recognized each of the subtypes. Production of IL-4 and IFN-γ was measured in serum samples collected from all five experiment groups 35 days after the prime immunization, using the commercially available mice cytokine ELISA kits (RD, USA).

### 2.6 Plaque reduction neutralization assay (PRNT)

Neutralization antibodies elicited in immunized mice were evaluated by PRNT as described previously [19]. Two fold serial dilutions of murine sera starting at 1:5 were tested. The percentage neutralization was calculated from the number of plaques obtained in the presence or absence of serum. The reciprocal of the highest serum dilution giving at least 50% neutralization was taken as the JEV neutralization titer.

### 2.7 Statistical analysis

All data analyses were conducted using SPSS biostatistics software (version 16.0, SPSS Inc., Chicago, IL, USA).

## 3. Results

### 3.1 Expressions of JEV prME-WT and three mutant proteins by Western blot analysis

**Figure 2:**
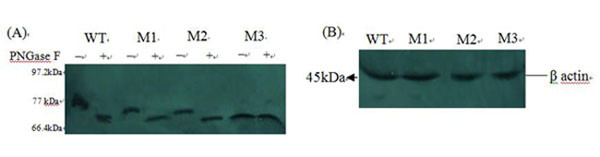
**Western blotting analysis of the expression of recombinant plasmids and β-actin.** (A)Samples were harvested from cell lysates at 48 h after transfection. prME-WT, prME-M1, PVAX-prME-M2, and PVAX-prME-M3 proteins were incubated with PNGase F (+) or buffer only (−). (B) β-actin was used as internal control.

Vero cells were transfected with pVAXI-prME-WT and the three plasmids encoding the mutant prME genes, pVAXI -prME-M1, pVAXI -prME-M2, and pVAXI -prME-M3. To determine whether the mobility shift observed for the JEV prME mutant proteins actually reflected the loss of glycans at the potential N-linked glycosylation sites, we treated or mock-treated immunoprecipitated lysates with PNGase F, which cleaves all types of N-linked glycans. The digestion of approximate 77 kDa prME-WT protein with PNGase F yielded a product of approximate 70 kDa, and the digestion of approximate 74 kDa prME- M1 protein with PNGase F yielded a product of approximate 70 kDa, whose migration pattern was invariably identical to those of prME-M2 protein. However, both the mock- and PNGase F-digested prME-M3 protein migrated at the same position as the PNGase F-digested prME-WT protein (Figure 2A). β-actin, a housekeeping gene with constant expression, was used as internal control (Figure 2B).

### 3.2 Antibody isotype profiles

**Figure 3:**
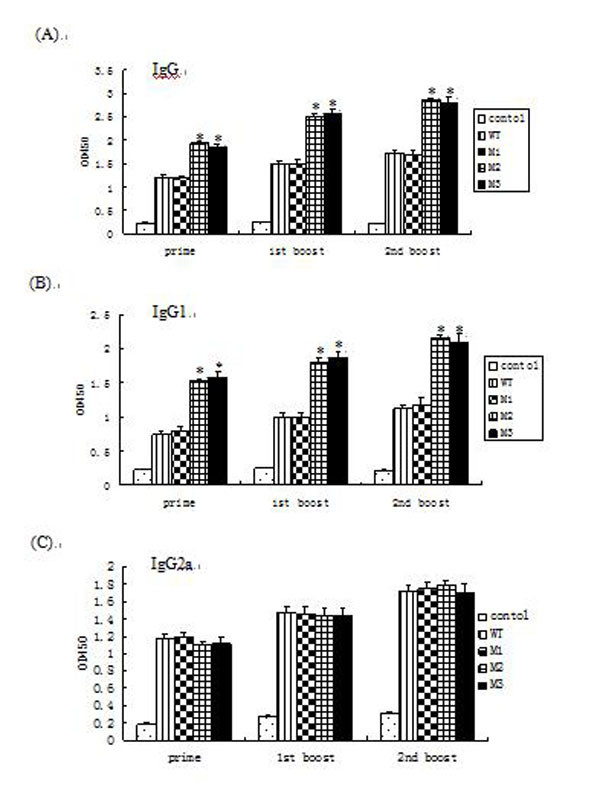
**Analysis of JEV-induced IgG isotypes**. Prime-boost-boost vaccinations were carried out. On day 7 (prime), day 21 (first boost) and day 35 (second boost) sera were taken and immunoglobulin isotypes were analyzed by ELISA. (A) IgG isotype response; (B) IgG1 isotype response; (C) IgG2a isotype response. Data presented as mean ± standard error for fourteen mice per group. ƟP < 0.05 compared with groups immunized with prME-WT in prime-boost-boost vaccinations.

The induction of prME-specific IgG antibodies in each of the animal groups was monitored by ELISA after immunization. JEV-prME specific antibody responses were induced following the first vaccination and increased following each booster. With respect to IgG and IgG1, vaccination with the prME-M2 and prME-M3 plasmids produced a stronger response than WT (Figure 3A,B). However, no significant differences were observed among the IgG2a antibody titers upon vaccination with any of the plasmids (Figure 3C). The strong IgG1 response present in animals immunized with prME-M2 and prME-M3 suggests a strong Th2-type immune response.

### 3.3 Cytokine response to prME mutants

**Figure 4:**
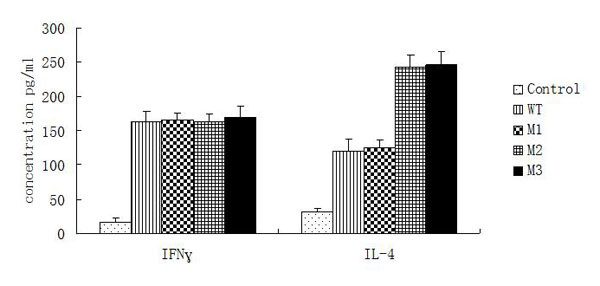
**Cytokine detection in splenocyte culture supernatants from vaccinated mice**. Splenocyte samples (n = 4 mice per group) isolated from the spleens of vaccinated mice on day 35 after the prime immunization were stimulated with purified JEV particles (10 ng/ml). After 72h, the supernatants were collected to examine the levels of the Th1-type cytokine IFN-γ and the Th2-type cytokine IL-4 using commercially available murine cytokine ELISA kits. Data presented as mean ± standard error for four mice per group.

Cellular immunity was evaluated by measuring the production of IFN-γ and IL-4 in spleen lymphocytes harvested 72 h after the second booster. As shown in (Figure 4), spleen lymphocytes from mice immunized with prME-M2 and prME-M3 secreted large amounts of IL-4, though the level of IFN-γ in these animals was not as elevated. Splenocytes from mice immunized with prME-WT and prME-M1, however, produced low levels of IL-4 and high levels of IFN-γ. Our results suggest that splenocytes from mice immunized with prME-M2 and prME-M3 predominantly induced a Th2-type immune response, because the levels of Th2-type cytokines (IL-4) were higher than those of Th1-type cytokines (IFN-γ). Thus, it appears that mutation of N154 in prME induces strong production of Th2 cytokines.

### 3.4 Production of neutralizing antibodies

Serum samples collected 35 days after the prime immunization were evaluated for the ability to neutralize JEV in plaque reduction neutralization assays. As shown in Table 2, the neutralization titer of serum from the prME-WT-immunized mice was very low, consistent with previous report. According to the previous report, in the process of JEV DNA vaccine, the low lever of neutralization titer could significantly produce the high protection rate after challenge [19]. However, the neutralization titers of serum from prME-M2- and prME-M3-immunized mice were higher than those induced by mice immunized with prME-WT and prME-M1. Neutralizing antibodies were not detected in controls. Our results suggest that mutating N154 in prME induces strong neutralizing antibody response.

**Table 2:** The neutralization titers of serum and protective immunity in vaccinated mice

DNA Vaccine	PRNT50	Survival rate (%)
pVAXⅠprME-WT	13 ± 4	90^b^
pVAXⅠprME-M1	15 ± 5	90^b^
pVAXⅠprME-M2	35 ± 9^a^	100^b^
pVAXⅠprME-M3	38 ± 8^a^	100^b^
pVAXⅠ(Control)	< 5	10

Data are shown as mean ± standard error (S.E.). Statistical analysis was performed using Student's t-test.

^a ^Significance (*P *< 0.05 to 13 ± 4 and 15 ± 5).

^b ^Significance (*P *< 0.05 to 10%).

### 3.5 Protection against JEV challenge in immunized mice

To investigate the degree to which the immunized mice were protected from JEV, all immunized mice were exposed to a lethal dose of the JEV strain NJ2008 and evaluated for their ability to survive the challenge. Mice immunized with prME-M2 or prME-M3 showed complete protection against JEV challenge. Nine out of ten mice immunized with prME-WT and prME-M1 survived JEV challenge, whereas only one mouse in the control group survived (Table 2).

## Discussion

Japanese encephalitis (JE) is a serious disease prevalent throughout Asia [20] and is transmitted to humans by mosquito bite [21]. Pigs are an important amplifier host for the virus [22] Vaccination of swine, therefore, can help prevent disease in humans [23].

DNA vaccines against JEV have shown great potential as preventative agents for their ability to elicit potent humoral and cytotoxic cellular immune responses against the plasmid-encoded protein in a broad range of hosts [24]. A previous study demonstrated that plasmids carrying the JEV prM and E genes can induce high NEUT antibodies and protective immunity in mice. Most importantly, the signal of prM is included in DNA vaccine, thus it is likely that viral antigens can be secreted from transfected cells and these DNA vaccines can induce high levels of immune responses [11]. In this study, we assessed the immunogenicity of several prME mutants to evaluate the potential of these mutants as DNA vaccines against JEV in swine.

The prM and E proteins of JEV are both exposed structural proteins. E is a major immunogenic antigen, and the prM is JEV induce protective immune additional components [25]. It has been reported that the prM proteins of flaviviruses can form natively folded structures independent of the E protein, form hetero-dimers with the E protein, and appear to act as folding chaperones for E protein [8]. In addition, the prME expression plasmids contain the signal of prM coding sequences for translocation into endoplasmic reticulum (ER), and the signal of prM can make the respective proteins are glycosylated or transported by the secretory pathway as supposed [11]. To investigate the effect of mutating putative N-glycosylation sites on the immunogenicity of prM and E, these proteins must maintain their native conformation.

Previous studies have demonstrated that N-linked glycans on the glycoproteins of many viruses play important roles in modulating the immune response. Removal of N-glycosylation sites in the simian immunodeficiency virus envelope protein and influenza virus hemagglutinin protein have been observed to limit the neutralizing antibody response [26], while mutation of N-linked glycans in human immunodeficiency virus type 1 (HIV-1) envelope protein appears to enhance the production of CTL [27], and deletion of glycans in hepatitis C virus E1 can enhance cellular or humoral immune responses [28]. The effects of the putative N-linked glycosylation sites in prM and E on the immune response to JEV are not known, however.

The JEV PrM protein contains one putative N-linked glycosylation site at N15, and the E protein also contains one putative N-linked glycosylation site, at N154. We demonstrate that immunization with the mutants prME-M2 and prME-M3, both of which contain the N154A mutation, induced a significantly enhanced antibody response, elevated IL-4 secretion levels, and full protection to lethal challenge of JEV compared to immunization with native prME, indicating that these mutations could elicit a stronger humoral immune response than the wild-type prME. We also demonstrate that mutating the N15 site (prME-M1) induces a humoral immune response comparable to that observed upon immunization with wild-type prME. This strongly suggests that mutating N15 in the prM glycoprotein does not strongly perturb the immune response to prME, but mutating N154 of the E glycoprotein does affect the immune response to prME.

Though DNA vaccines generally induce a stronger Th1 immune response, producing elevated levels of IFN-γ and IgG2a, the immune responses induced by DNA vaccines need to be improved. Our results show that a single mutation, N154A, significantly enhances the humoral immune response. We propose, therefore, that this highly immunogenic mutant could serve as a swine vaccine against JEV and should be further optimized for this purpose.

## Abbreviations

JEV: Japanese encephalitis virus; DNA: Deoxyribonucleic Acid; TBEV: Tick-borne encephalitis virus; WNV: West Nile virus; IgG: Immunoglobulin G; cDNA: complementary Deoxyribonucleic Acid; PCR: Polymerase Chain Reaction; PNGase F: Peptide N-Glycosidase F; β-actin: beta-actin; IFN-γ: IFN-gamma.

## Competing interests

The authors declare that they have no competing interests.

## Authors' contributions

YZ, RC and PC participated in the design and conducted the majority of the experiments in the study and drafted the manuscript. JG contributed to the interpretation of the findings and revised the manuscript. All authors read and approved the final manuscript.
